# Extraction and reimplantation of cardiac implantable electronic devices in patients with non-surgically treated infective endocarditis: a nationwide cohort study

**DOI:** 10.1093/ehjopen/oeag008

**Published:** 2026-01-21

**Authors:** Mohammed Bakir Ahmad Lafta, Amna Alhakak, Lauge Østergaard, Niels Eske Bruun, Anne-Christine Ruwald, Melanie Vuong Le, Berit Philbert, Michael Vinther, Peter Godsk Jørgensen, Eva Havers-Borgersen, Louise Kruse Jensen, Jonas Agerlund Povlsen, Jens Cosedis Nielsen, Jens Brock Johansen, Marianne Voldstedlund, Claus Moser, Henning Bundgaard, Lars Køber, Emil Fosbøl

**Affiliations:** Department of Cardiology, Copenhagen University Hospital—Rigshospitalet, Blegdamsvej 9, 2100 Copenhagen, Denmark; Department of Cardiology, Copenhagen University Hospital—Rigshospitalet, Blegdamsvej 9, 2100 Copenhagen, Denmark; Department of Cardiology, Copenhagen University Hospital—Rigshospitalet, Blegdamsvej 9, 2100 Copenhagen, Denmark; Department of Cardiology, Zealand University Hospital, Sygehusvej 10, 4000 Roskilde, Denmark; Department of Clinical Medicine, University of Copenhagen, Blegdamsvej 3B, 2200 Copenhagen, Denmark; Department of Cardiology, Copenhagen University Hospital—Rigshospitalet, Blegdamsvej 9, 2100 Copenhagen, Denmark; Department of Cardiology, Zealand University Hospital, Sygehusvej 10, 4000 Roskilde, Denmark; Department of Cardiology, Zealand University Hospital, Sygehusvej 10, 4000 Roskilde, Denmark; Department of Cardiology, Copenhagen University Hospital—Rigshospitalet, Blegdamsvej 9, 2100 Copenhagen, Denmark; Department of Cardiology, Copenhagen University Hospital—Rigshospitalet, Blegdamsvej 9, 2100 Copenhagen, Denmark; Department of Cardiology, Copenhagen University Hospital—Herlev and Gentofte, Borgmester Ib Juuls Vej 1, 2730 Herlev, Denmark; Department of Cardiology, Copenhagen University Hospital—Rigshospitalet, Blegdamsvej 9, 2100 Copenhagen, Denmark; Department of Cardiology, Zealand University Hospital, Sygehusvej 10, 4000 Roskilde, Denmark; Department of Veterinary and Animal Sciences, Section of Pathobiological Sciences, University of Copenhagen, 1870 Frederiksberg C, Denmark; Department of Cardiology, Aarhus University Hospital, Palle Juul-Jensens Boulevard 99, 8200 Aarhus N, Denmark; Department of Cardiology, Aarhus University Hospital, Palle Juul-Jensens Boulevard 99, 8200 Aarhus N, Denmark; Department of Clinical Medicine, Aarhus University, Nordre Ringgade 1, 8000 Aarhus C, Denmark; Department of Cardiology, Odense University Hospital, J.B. Winsløws Vej 4, 5000 Odense C, Denmark; Department of Data Integration and Analysis, Statens Serum Institute, Artillerivej 5, 2300 Amager, Copenhagen, Denmark; Department for Immunology and Microbiology, University of Copenhagen, Blegdamsvej 3B, 2200 Copenhagen, Denmark; Department of Clinical Microbiology, Copenhagen University Hospital—Rigshospitalet, Blegdamsvej 9, 2100 Copenhagen, Denmark; Department of Cardiology, Copenhagen University Hospital—Rigshospitalet, Blegdamsvej 9, 2100 Copenhagen, Denmark; Department of Clinical Medicine, University of Copenhagen, Blegdamsvej 3B, 2200 Copenhagen, Denmark; Department of Cardiology, Copenhagen University Hospital—Rigshospitalet, Blegdamsvej 9, 2100 Copenhagen, Denmark; Department of Clinical Medicine, University of Copenhagen, Blegdamsvej 3B, 2200 Copenhagen, Denmark; Department of Cardiology, Copenhagen University Hospital—Rigshospitalet, Blegdamsvej 9, 2100 Copenhagen, Denmark; Department of Clinical Medicine, University of Copenhagen, Blegdamsvej 3B, 2200 Copenhagen, Denmark

**Keywords:** *Cardiac implantable electronic device*, *Pacemaker*, *Implantable cardioverter–defibrillator*, *Infective endocarditis*, *Reimplantation*, *Outcomes*

## Abstract

**Aims:**

Cardiac implantable electronic device (CIED)-related infective endocarditis (IE) is associated with morbidity and mortality. Current guidelines recommend complete CIED extraction; however, the optimal timing for reimplantation remains uncertain. We aimed to evaluate CIED reimplantation rates, and outcomes in patients with non-surgically treated IE who underwent CIED extraction.

**Methods and results:**

We included all Danish residents ≥18 years diagnosed with first-time IE (2010–2021), with a pre-existing CIED, who underwent CIED extraction without concurrent valve surgery. Data were obtained from Danish nationwide registries. The primary variable of interest was CIED reimplantation within 90 days after extraction. Reimplantation rates were described, and reimplantation status was used to stratify patients for analysis of secondary outcomes, including recurrent IE-related bacteraemia and all-cause mortality within 6 months. Among 661 patients with CIED extraction due to IE, 396 (59.9%) underwent reimplantation within 3 months, with a median of 29 days (IQR:19–42 days). There was no significant difference in the 6 month cumulative incidence of recurrent bacteraemia with IE (2.5% [95% CI:0.95–5.5] vs. 1.8% [95% CI:0.8–3.6] *P* = 0.55) or mortality (11.2% [95% CI:7.0–16.4] vs. 7.0% [95% CI:4.7–10.0]; *P* = 0.11) between non-reimplanted and reimplanted patients.

**Conclusion:**

In IE patients who underwent CIED extraction, 60% of patients were reimplanted within 3 months with substantial variation in timing. No significant differences in outcomes were found by reimplantation status.

## Graphical Abstract

**Graphical Abstract:**
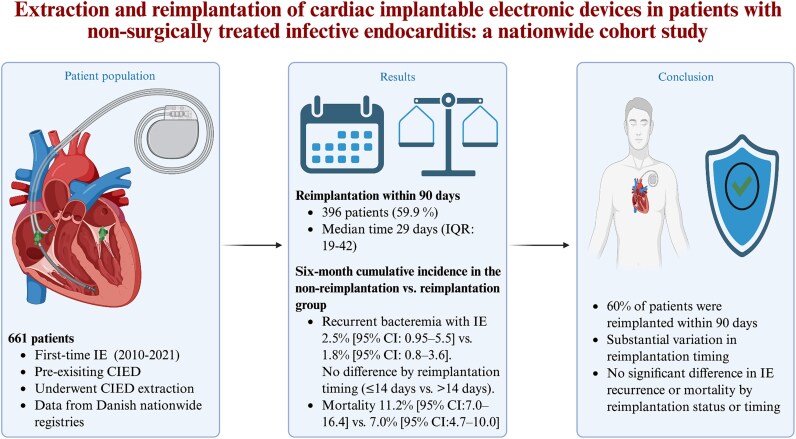

## Introduction

The increasing number of cardiac implantable electronic device (CIED) implantations over the years has been paralleled by a rising incidence of CIED-related infective endocarditis (CIED–IE).^1^ This condition is associated with significant mortality, morbidity, severe complications, increased hospitalizations, and higher healthcare costs.^2,3^ The rising incidence of CIED-related IE may be driven by an ageing population, broader device indications, and the growing procedural complexity of implanting multiple leads and performing generator replacements.^4–6^ Although these complicating factors exist, it is important to acknowledge that CIEDs have significantly improved the treatment and prognosis of many cardiac conditions.^2,4,7^

According to current guidelines, the optimal management of CIED infections, whether classified as definite or possible, often involves complete CIED extraction, combined with extended antibiotic treatment.^2,8^ However, some patients continue to require CIED support, making the timing of reimplantation a critical consideration. As of now, precise recommendations on the timing of reimplantation following infection-related device extraction is lacking. Multiple factors influence this decision, including pacing indication, lead dwell time, the type and severity of infection, inflammatory marker levels, and the patient’s clinical response to treatment.^9^

In view of the rising incidence^10^ and serious consequences of CIED-related IE, understanding reimplantation patterns and associated outcomes is essential. Therefore, this study aimed to evaluate reimplantation rates and reimplantation timing among patients with non-surgically treated IE who underwent CIED extraction. Additionally, we set out to investigate the factors associated with reimplantation, as well as associated outcomes, including recurrent bacteraemia with and without IE, IE readmission, and all-cause mortality, stratified by reimplantation status.

## Methods

### Data source

Every citizen in Denmark is assigned a unique and permanent civil registration number making it possible to link nationwide administrative registries. In this nationwide study, we collected data from the Danish National Patient Registry (DNPR),^11^ the Danish National Prescription Registry,^12^ the Danish population registry,^13^ and the Danish Microbiology Database.^14^ The DNPR registry contains information on every hospital admission and outpatient contact since 1977, with diagnoses coded by physicians. Until 1994, diagnostic coding followed the International Classification of Diseases, Eighth Revision (ICD-8), transitioning thereafter to the Tenth Revision (ICD-10). The DNPR also provides data on surgical procedures based on the Nordic Medico-Statistical Committee Classification of Surgical Procedures (NCSP). The Danish Microbiology Database provides data from all Danish departments of clinical microbiology since 2010. The Danish National Prescription Registry contains data on all filled prescriptions at Danish pharmacies, classified according to Anatomical Therapeutic Chemical (ATC) codes. Demographic variables such as sex, date of birth, date of death, and migration status were provided from the Danish Civil Registration System. All four registries are validated and of high quality.^11–14^

### Study population

The main study population included all Danish residents aged ≥18 years who were hospitalized with first-time IE from 1 January 2010 to 31 December 2021 and who were non-surgically treated, defined as no heart valve surgery during IE admission (see Supplementary material online, *Table S1* for NCSP codes of heart valve surgery). Patients with IE were identified based on inpatient primary or secondary diagnoses using the following ICD-10 codes: acute endocarditis (DI339), acute or subacute IE (DI330), endocarditis, unspecified, in diseases classified elsewhere (DI398), and endocarditis unspecified (DI38). The accuracy of the IE diagnosis in the DNPR has been previously validated with a high positive predictive value (PPV) of 90% for admission ≥14 days, based on the ICD-10 codes.^15,16^ Therefore, we included patients who were hospitalized for ≥14 days or those who died within 14 days during IE admission.

Only patients with a pre-existing CIED [i.e. pacemakers (PM), implantable cardioverter defibrillators (ICD), and cardiac resynchronization therapy with or without defibrillator (CRT-D or CRT-*P*)] who underwent CIED extraction during their IE admission were included in the study cohort. We included data on the most recent CIED procedure from the DNPR, recorded at any time prior to IE admission (see Supplementary material online, *Table S2* for NCSP codes of CIED procedures and CIED extraction). The PPVs for the procedure codes of CIEDs in the DNPR have been validated with a high PPV of 100%.^17^

We used the Danish Microbiology Database to identify a possible microbiological cause of IE for the included patients. If multiple blood culture results were available, a bacterial hierarchy was performed based on the relevance to IE: (i) highest priority included *Staphylococcus aureus*, *Enterococcus species*, *Streptococcus species*, and HACEK group organisms [*Haemophilus* species (not including *Haemophilus influenzae*), *Aggregatibacter* species, *Cardiobacterium* species, *Eikenella* species, *Kingella* species]; (ii) intermediate priority included coagulase-negative staphylococci (CoNS); (iii) low priority covered other bacterial findings such as rare bacteria and bacteria not identified at the species level; and (iv) lowest priority was assigned to blood culture-negative results. Due to the low number of HACEK organisms, this group was consequently included in the third category, covering other bacterial findings.

### Patient characteristics, comorbidity, and pharmacotherapy

Comorbidities were registered based on inpatient and outpatient diagnoses recorded as primary or secondary diagnoses any time before IE admission, using the ICD-8 and ICD-10 codes (see Supplementary material online, *Table S3* for ICD-8 and ICD-10 diagnosis codes). Hypertension was defined, as previously validated, based on redeemed prescriptions for a combination of at least two antihypertensive medications.^18^ Diabetes was defined, as previously described, based on inpatient or outpatient contacts according to ICD-8 and ICD-10 codes or on redeemed prescriptions for antidiabetic drugs within 6 months prior to IE admission.^19,20^

Concomitant pharmacotherapy was identified based on ATC codes for redeemed prescriptions within 6 months prior to the IE admission (see Supplementary material online, *Table S4* for ATC codes).

Included patients were categorized into frailty groups at the time of IE admission using the previously validated Hospital Frailty Risk Score.^21^ For every patient, a score was calculated based on data from hospital admissions within 10 years prior to their IE admission. Patients were categorized into three groups based on frailty risk: low (0–4 points), intermediate (5–15 points), and high (>15 points). Patients with a score of ≥5 points were considered as frail.

Diagnoses timely associated with initial CIED implantation were defined within 6 months prior to initial CIED procedure. Secondary prevention was defined as cardiac arrest, ventricular tachycardia (VT), or ventricular fibrillation (VF) within 6 months prior to initial CIED procedure in patients with ICD or CRT-D.

### Study outcome

The primary variable of interest was CIED reimplantation within 90 days after extraction (see Supplementary material online, *Table S5* for NCSP codes). The secondary outcomes were IE readmission (regardless of microbiological aetiology), IE relapse, recurrent bacteraemia with IE, recurrent bacteraemia without IE (all defined as involving the same microbiological aetiology as the first IE episode), and all-cause mortality within 6 months. All secondary outcomes were analysed stratified by reimplantation status, and recurrent bacteraemia with IE was also stratified by reimplantation timing (≤14 days vs. >14 days). The 14 day cut-off was selected based on clinical rationale, as current guidelines recommend reimplantation only after signs and symptoms of infection have abated and blood cultures have been negative for ≥72 h (or ≥2 weeks in the presence of vegetations).^8^ The follow-up for the IE-related secondary outcomes began the day after IE discharge, whereas the follow-up for all-cause mortality began 90 days post-extraction. This ensured that all patients had the opportunity for reimplantation, thereby reducing survival bias when comparing reimplanted and non-reimplanted patients. Only patients who survived until IE discharge or 90 days post-extraction were included in the secondary outcome analyses.

### Statistics

For comparison of baseline characteristics, the study population was stratified according to CIED reimplantation status. The *χ*^2^ test was applied for categorical variables, while the Wilcoxon test was used for continuous variables. Categorical variables are reported as frequencies with percentages, whereas continuous variables are reported as medians with interquartile ranges (IQRs).

The cumulative incidences of CIED reimplantation after the extraction date were calculated using the Aalen–Johansen estimator, accounting for death as competing event. Cause-specific Cox regression analyses were performed to assess factors associated with CIED reimplantation, presented as hazard ratios (HRs) with 95% confidence intervals (CIs). The model was adjusted for sex, age categories (i.e. <70 years, 70–80 years, and >80 years), calendar year of IE admission, time since the most recent CIED procedure, CIED type, diagnoses timely associated with initial CIED implantation such as secondary prevention, second- and third-degree AV block, sick sinus syndrome (SSS), and history of comorbidities such as ischaemic heart disease, atrial fibrillation, stroke, chronic obstructive pulmonary disease (COPD), cancer, diabetes, frailty risk groups, and previous left-sided prosthetic heart valve surgery.

Furthermore, a histogram was created to visualize the time intervals from CIED extraction to reimplantation, while a transition plot was created to illustrate the change in CIED types from before extraction to after reimplantation.

All model assumptions for the cause-specific Cox regression were met, including proportional hazards and no significant interactions between CIED type and other clinically relevant variables.

All statistical analyses were performed with SAS (version 9.4, Cary, NC, USA) on the secure servers of Statistics Denmark. A two-sided *P*-value < 0.05 was considered statistically significant.

### Ethics approval

In Denmark, ethics committee approval is not required for registry-based studies. All personal identifiers were pseudo anonymized, and microdata with observations ≤3 patients were not reported, in accordance with Statistics Denmark’s anonymization guidelines. The study data were provided by Statistics Denmark and approved by the responsible institute, The Capital Region of Denmark (approval number P-2019–191), in accordance with the General Data Protection Regulation.

## Results

**Figure 1:**
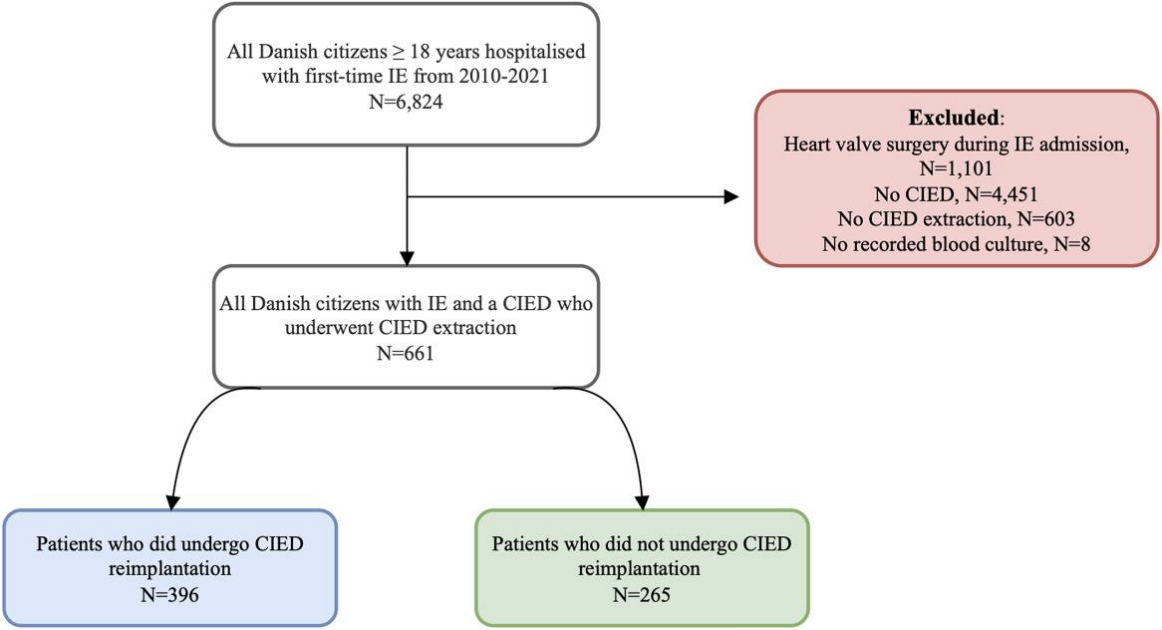
**Study population selection.** IE, Infective endocarditis; CIED, cardiac implantable electronic device.

A total of 6824 patients were diagnosed with first-time IE between 1 January 2010 and 31 December 2021. Of these, 5723 (83.9%) were treated non-surgically (*Figure 1*), and 1272 (18.6%) of them had a CIED. Among patients with non-surgically managed IE and a CIED, 669 (52.6%) underwent CIED extraction. Eight patients (0.6%) did not have a recorded blood culture and were excluded, leaving 661 patients in the primary study population, corresponding to 52.0% of all patients with a CIED. Among these, 396 (59.9%) received CIED reimplantation, while 265 (40.1%) did not. Within the primary population, 94 patients (14.2%) died within 90 days after extraction. The remaining 567 patients who survived 90 days after extraction comprised the cohort for analyses of all-cause mortality, while 596 patients who survived until IE discharge comprised the population for IE-related outcomes. Among those who survived 90 days post-extraction, 386 (68.1%) received CIED reimplantation, while 181 (31.9%) did not.

### Patient characteristics

The baseline characteristics, stratified according to CIED reimplantation status, are summarized in *Table 1*. The median age of the total study population was 73.7 years ([IQR]: 66.0–79.6), and 520 (78.7%) were males. Patients who underwent CIED reimplantation had similar age compared to those who did not (median age: 73.4 years [IQR: 66.0–79.5] vs. 74.4 years [IQR: 66.0–79.6]) (*P* = 0.55). The median frailty score was significantly lower in the reimplantation group, with a median frailty score of 5.6 [IQR: 2.1–9.7], compared to 6.3 [IQR: 2.6–11.7] in the non-reimplantation group (*P* = 0.02). The time from the most recent CIED procedure to IE admission was borderline significantly shorter in reimplanted patients (median: 1.5 years [IQR: 0.3–3.6]) compared to non-reimplanted patients (median: 1.8 years [IQR: 0.4–4.4]) (*P* = 0.051).

**Table 1:** Baseline characteristics according to status of reimplantation

Variable	Total (*n* = 661)	No reimplantation (*n* = 265)	Reimplantation (*n* = 396)	*P*-value
Sex (male)	520 (78.7)	197 (74.3)	323 (81.6)	0.03
Age (median [IQR])	73.7 [66.0, 79.6]	74.4 [66.0, 79.6]	73.4 [66.0, 79.5]	0.55
**Age group**				
<70 years	244 (36.9)	96 (36.2)	148 (37.4)	0.93
70–80 years	267 (40.4)	107 (40.4)	160 (40.4)	
>80	150 (22.7)	62 (23.4)	88 (22.2)	
**Frailty score group**				
Low	287 (43.4)	103 (38.9)	184 (46.5)	0.13
Intermediate	287 (43.4)	122 (46.0)	165 (41.7)	
Severe	87 (13.2)	40 (15.1)	47 (11.9)	
Frailty score (median [IQR])	6.0 [2.3, 10.4]	6.3 [2.6, 11.7]	5.6 [2.1, 9.7]	0.02
**Microbiological aetiology**				
*Staphylococcus aureus*	219 (33.1)	96 (36.2)	123 (31.1)	0.23
*Enterococcus* spp.	85 (12.9)	38 (14.3)	47 (11.9)	
CoNS	65 (9.8)	29 (10.9)	36 (9.1)	
*Streptococcus* spp.	79 (12.0)	29 (10.9)	50 (12.6)	
Negative blood culture	138 (20.9)	44 (16.6)	94 (23.7)	
Other	75 (11.4)	29 (10.9)	46 (11.6)	
**CIED type**				
Cardiac resynchronization therapy with defibrillator	79 (12.0)	26 (9.8)	53 (13.4)	0.38
Cardiac resynchronization therapy with pacemaker	37 (5.6)	18 (6.8)	19 (4.8)	
Implantable cardioverter–defibrillator	152 (23.0)	64 (24.2)	88 (22.2)	
Pacemaker	393 (59.5)	157 (59.3)	236 (59.6)	
Time from the most recent CIED procedure to IE admission, years (median [IQR])	1.6 [0.3, 3.9]	1.8 [0.4, 4.4]	1.5 [0.3, 3.6]	0.051
Time from the index CIED procedure to IE admission, years (median [IQR])	3.8 [0.9, 8.1]	3.8 [1.0, 7.9]	3.8 [0.9, 8.3]	0.84
Prior left-sided prosthetic heart valve surgery	98 (14.8)	41 (15.5)	57 (14.4)	0.70
**History of comorbidity**				
Ischaemic heart disease	374 (56.6)	157 (59.3)	217 (54.8)	0.26
Heart failure	355 (53.7)	151 (57.0)	204 (51.5)	0.17
Atrial fibrillation	341 (51.6)	132 (49.8)	209 (52.8)	0.45
Stroke	101 (15.3)	38 (14.3)	63 (15.9)	0.58
Hypertension	485 (73.4)	193 (72.8)	292 (73.7)	0.80
Diabetes	234 (35.4)	104 (39.3)	130 (32.8)	0.09
Chronic obstructive pulmonary disease	109 (16.5)	48 (18.1)	61 (15.4)	0.36
Cancer	131 (19.8)	59 (22.3)	72 (18.2)	0.20
Chronic renal disease	131 (19.8)	56 (21.1)	75 (18.9)	0.49
Liver disease	37 (5.6)	22 (8.3)	15 (3.8)	0.01
**Concomitant pharmacotherapy**				
Cholesterol-lowering drugs	383 (57.9)	146 (55.1)	237 (59.9)	0.23
Beta-blockers	424 (64.2)	166 (62.6)	258 (65.2)	0.51
Calcium channel blockers	133 (20.1)	41 (15.5)	92 (23.2)	0.01
Renin angiotensin system inhibitors	404 (61.1)	158 (59.6)	246 (62.1)	0.52
Acetylsalicylic acid	220 (33.3)	96 (36.2)	124 (31.3)	0.19
Adenosine diphosphate receptor inhibitors	96 (14.5)	38 (14.3)	58 (14.7)	0.91
Anticoagulants	334 (50.5)	126 (47.6)	208 (52.5)	0.21
Immunosuppressive	11 (1.7)	4 (1.5)	7 (1.8)	0.80

No significant differences in comorbidity status were identified between the two groups, except that diagnosed liver disease was more common in the non-reimplantation group than in the reimplantation group (8.3 vs. 3.8%, *P* = 0.01).

Pacemakers were the most prevalent CIED type across the entire cohort (59.5%), with no significant differences in the distribution of CIED types between the reimplantation and non-reimplantation group. However, the indications, defined as the diagnoses timely associated with the initial CIED implantation, differed significantly between groups (*Table 2*); atrioventricular block (AVB2/3) was significantly more frequent in the reimplantation group (32.8 vs. 18.5%, *P* < 0.001), as was secondary prevention among patients with an ICD or CRT-D (70.2 vs. 29.8%, *P* = 0.02), whereas SSS was significantly more frequent in the non-reimplantation group (22.6 vs. 14.7%, *P* = 0.009). In a supplementary analysis, atrioventricular block (AVB2/3) was more frequent among patients aged >80 years (37.3%) compared with those aged 70–80 years (22.1%) and <70 years (26.2%). In contrast, ICD therapy was less common among patients aged >80 years (7.3 vs. 22.9% and 32.8%), as was a history of VT/VF (3.3 vs. 7.1% and 11.5%).

**Table 2:** Diagnoses timely associated with initial CIED implantation

Variable	Total (*n* = 661)	No reimplantation (*n* = 265)	Reimplantation (*n* = 396)	*P*-value
Cardiac arrest	37 (5.6)	12 (4.5)	25 (6.3)	0.33
Ventricular tachycardia and ventricular fibrillation	52 (7.9)	16 (6.0)	36 (9.1)	0.15
First-degree atrioventricular block	≤3	—	—	—
Second-degree or third-degree atrioventricular block	179 (27.1)	49 (18.5)	130 (32.8)	<0.001
Branch and fascicular block	14 (2.1)	7 (2.6)	7 (1.8)	0.44
Sick sinus syndrome (SSS)^a^	118 (17.9)	60 (22.6)	58 (14.7)	0.009
Heart failure	230 (34.8)	101 (38.1)	129 (32.6)	0.14
Ischaemic heart disease	194 (29.4)	90 (34.0)	104 (26.3)	0.03
Ion channel disorders ^b^	<3	—	—	—
Syncope ^c^	70 (10.6)	34 (12.8)	36 (9.1)	0.13

^a^Includes sinus bradycardia, sinus arrest and sinoatrial block.

^b^Ion channel disorders include Brugada syndrome, long QT syndrome, and short QT syndrome.

^c^Covers the diagnosis of Vasovagal syncope and carotid sinus syncope.

Supplementary material online, *Figure S1*, illustrates microbial aetiology according to reimplantation status. *Staphylococcus aureus* was the most commonly identified bacteria overall. There were no significant differences in microbial aetiology between the reimplantation and non-reimplantation groups.

### CIED reimplantation

**Figure 2:**
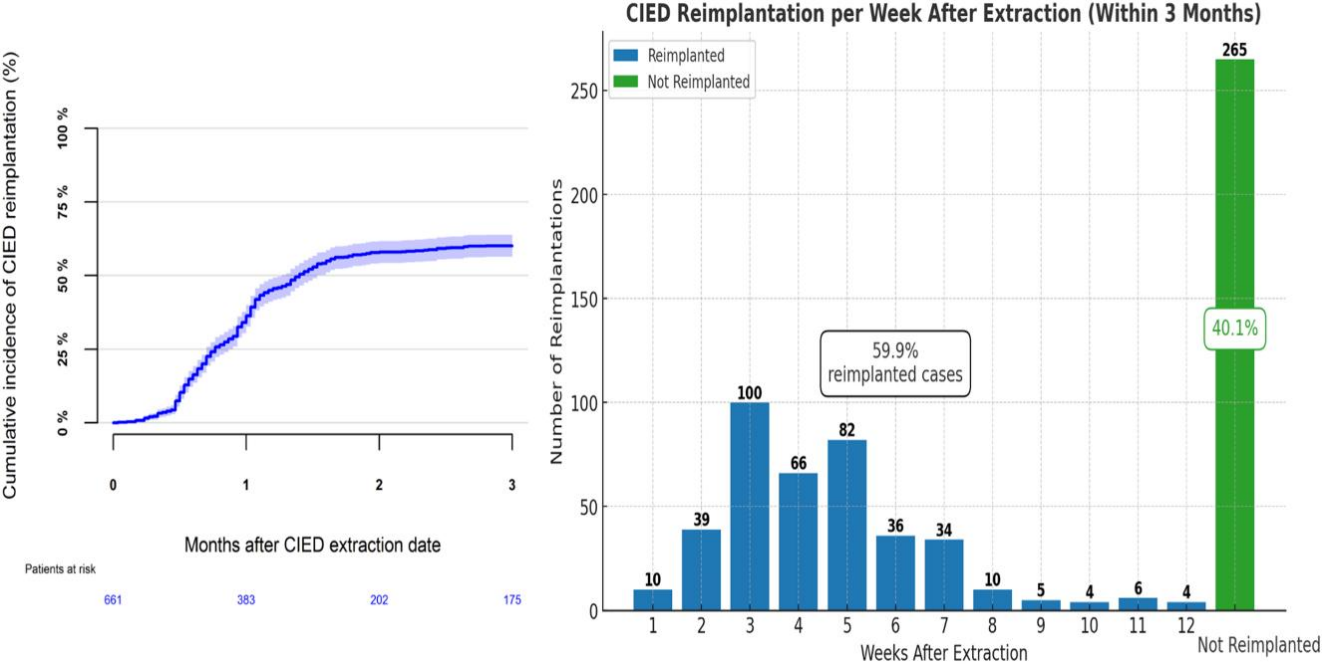
Cumulative incidence (*A*) and histogram (*B*) of reimplantation within 90 days after CIED extraction.

**Figure 3:**
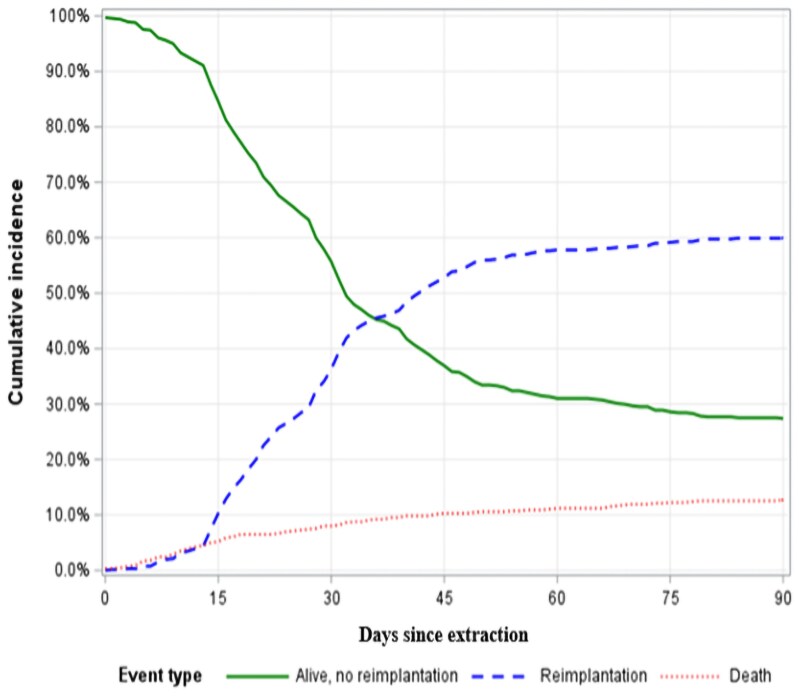
Cumulative incidence proportions of reimplantation, survival without reimplantation, and death within 90 days after CIED extraction. Ten patients were reimplanted and died within the 90 days.

The median time to reimplantation was 29 days [IQR: 19.0–42.0] after extraction. *Figure 2A* illustrates the cumulative incidence curve of reimplantation, with the cumulative incidence reaching 60.1% [95% CI: 56.3–63.8%]. A histogram of time from extraction to reimplantation (*Figure 2B*) shows that 62.6% of reimplantations occurred within 3–5 weeks after extraction. To provide a broader overview of patient outcomes, *Figure 3* illustrates the cumulative incidence proportions of reimplantation, survival without reimplantation, and death within 90 days after extraction. At Day 90, 181 (27.4%) were alive without reimplantation, and 84 (12.7%) had died. Among the reimplanted patients, 10 died within the 90 day period.

**Figure 4:**
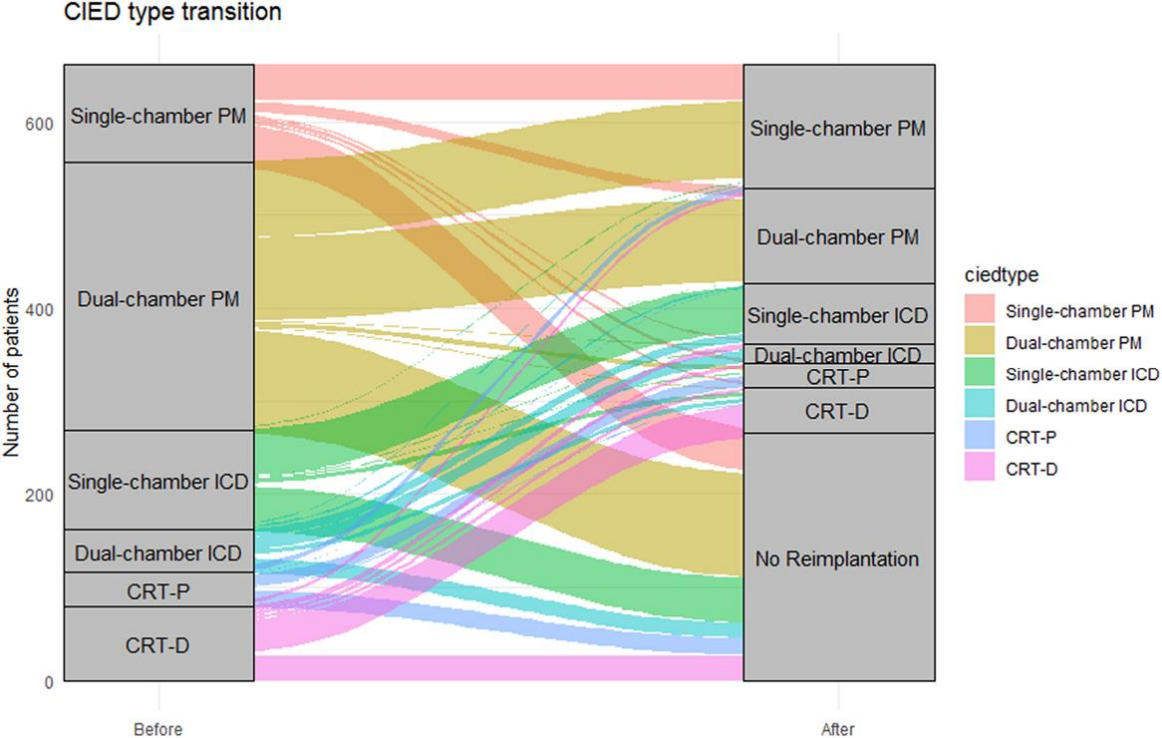
Transitions between initial and reimplanted CIED types. CRTD, cardiac resynchronization therapy with defibrillator. CRTP, cardiac resynchronization therapy with pacemaker. Dual-chamber ICD, dual-chamber implantable cardioverter–defibrillator. Single-chamber ICD, single-chamber implantable cardioverter–defibrillator. Dual-chamber PM, dual-chamber pacemaker. Single-chamber PM, single-chamber pacemaker.

*Figure 4* illustrates the transition of CIED types from the most recent CIED procedure prior to extraction to the reimplanted device. Compared to pre-extraction, the proportion of single-chamber pacemakers increased (104 before vs. 132 after), while the use of dual-chamber pacemakers declined (289 before vs. 102 after). In contrast, the proportion of CRT-*P*, CRT-D, and ICD devices did not change substantially. Among the reimplanted patients, 58 (14.7%) received a leadless pacemaker or a subcutaneous ICD (S-ICD).

### Factors associated with reimplantation

Factors associated with CIED reimplantation in the cause-specific Cox regression model are shown in Supplementary material online, *Figure S2*. The atrioventricular block (AVB2/3) as the diagnosis timely associated with the initial CIED implantation was significantly associated with a higher rate of reimplantation, with a HR of 1.53 [95% CI: 1.18–1.97]. In contrast, SSS, when present as the implantation-related diagnosis, was significantly associated with a lower rate of reimplantation (HR: 0.71 [95% CI: 0.52–0.97]). Additionally, atrial fibrillation as a comorbidity and age over 80 years, compared to those under 70 years, were also associated with a significantly higher rate of reimplantation, with a HR of 1.32 [95% CI: 1.07–1.62]) and 1.35 [95% CI: 1.02–1.80], respectively.

### All-cause mortality and infective endocarditis-related outcomes after cardiac implantable electronic device extraction

The non-reimplantation and reimplantation group had similar cumulative incidence rates of IE readmissions (5.0% [95% CI: 2.6–8.7] (*n* = 10) vs. (3.6% [95% CI: 2.1–5.8] (*n* = 14); *P* = 0.41), relapse IE (≤3 observations in both groups), recurrent bacteraemia with IE (2.5% [95% CI: 0.95–5.5] (*n* = 5) vs. 1.8% [95% CI: 0.8–3.6] (*n* = 7); *P* = 0.55), recurrent bacteraemia without IE (≤3 observations in the non-reimplantation group vs. 1.6% [95% CI: 0.7–3.2] (*n* = 6) in the reimplantation group). The 6 month cumulative incidence of mortality was also similar between the non-reimplantation and reimplantation group 11.2% [95% CI: 7.0–16.4] (*n* = 19) vs. (7.0% [95% CI: 4.7–10.0] (*n* = 26); *P* = 0.11). There was no significant difference in recurrent bacteraemia with IE between patients reimplanted ≤14 days (≤3 observations) vs. >14 days (1.5% [95% CI: 0.6–3.3], *n* = 5), as well as between those reimplanted ≤29 days (2.3% [95% CI: 0.9–4.9], *n* = 5) vs. >29 days (≤3 observations).

## Discussion

In this nationwide study of patients with IE who underwent CIED extraction without valve surgery, we identified three major findings. First, approximately 60% of the study population underwent CIED reimplantation within 3 months after extraction, with a median time to reimplantation of 29 days. Second, atrioventricular block (AVB2/3), atrial fibrillation as a comorbidity, and age over 80 years were associated with significantly higher rates of reimplantation, whereas SSS was associated with significantly lower reimplantation rate. Additionally, the reimplantation group had a lower median frailty score than the non-reimplantation group. Third, IE readmissions, relapse IE, recurrent bacteraemia with and without IE, and all-cause mortality rates were similar between the two groups and by timing of reimplantation.

### CIED reimplantation

According to the 2023 ESC guidelines on managing IE,^8^ CIED reimplantation should be performed at a site distant from the previous generator, as late as possible and only once signs and symptoms of infection have resolved. Blood cultures should be negative for at least 72 h in patients without vegetations and for at least 2 weeks if vegetations are present. The recommendation for reimplantation is classified as Class I, Level of Evidence C, indicating expert consensus but limited clinical data. This is broadly consistent with the 2019 EHRA international consensus document,^2^ which places particular emphasis on the need for individualized decision-making, considering the clinical condition, infection severity, and ongoing device indications.

The observed extraction rate of 52.6% among patients with a CIED may partly reflect diagnostic uncertainty in patients with possible IE. In such cases, current ESC guidelines recommend device extraction as a Class IIa indication, allowing for a more conservative approach based on individual assessment. This may have contributed to the size and characteristics of the study population included in our analysis. Additionally, our study is based on nationwide data covering all hospital types, providing a more complete and unselected population. Previous studies^22–25^ have often been conducted in highly specialized tertiary centres, where reported extraction rates have varied widely (11.5–79.7%), potentially reflecting other extraction strategies.

In this nationwide study, the overall reimplantation rate of ∼60% is consistent with previous reports, which have documented reimplantation rates ranging from 57.7 to 80.7%.^5,26,27^ These findings indicate that the majority of patients underwent CIED reimplantation within 3 months post-extraction with variations across different study populations and clinical settings. Notably, the study^5^ with the reimplantation rate closest to ours (57.7%) was based on a multicentre registry including 434 patients across 10 centres, similar to our nationwide data. In contrast, the other studies were based on data from single centres, potentially limiting the generalizability of their results due to selection bias or specific local treatment strategies.^28^

In our cohort, more than half (62.6%) of reimplantations occurred within 3–5 weeks after extraction, with a median time of 29 days. According to the 2023 ESC guidelines on managing IE,^8^ antibiotic treatment is generally recommended for 2–6 weeks, depending on whether the infection involves a native or prosthetic valve, whether it is isolated left- or right-sided IE, as well as the causative pathogen. This recommended duration is based on empirical evidence, as no randomized data have been published.

The lack of a significant association between CIED type (ICD, CRT-*P*, and CRT-D) and reimplantation should be interpreted with caution, as this may reflect limited statistical power rather than a true absence of difference. It is noteworthy that reimplantation was not performed in a substantial proportion of patients across all CIED types, suggesting that continued device therapy was not always clinically necessary. These findings may highlight the importance of reassessing implantation and replacement indications, particularly in patients with an increased risk of CIED-related infection.

The transition from dual-chamber to single-chamber pacemakers likely reflects a principle of using the simplest effective CIED type. Minimizing hardware complexity by reducing the number of leads and chambers not only aligns with best practice but may also lower procedural risks,^29,30^ particularly in a frail patient group with a history of IE. Another possible explanation is the increased use of leadless devices, which are predominantly single-chamber systems and are considered safer following CIED infections due to their lower complication rates, including reduced reinfection risk.^31,32^Additionally, fewer patients may now meet the criteria for dual-chamber pacing. Meanwhile, differences in the use of CRT-*P*, CRT-D, and ICD devices were small and not statistically significant, suggesting that the trend primarily affected conventional pacemaker therapy.

A key challenge in the reimplantation process is determining the optimal timing to balance infection clearance with the patient’s cardiac dependence on CIEDs. This ‘critical decision window’ requires clinicians to carefully evaluate both the type of device to reimplant and the most appropriate timing, aiming to minimize the risk of recurrent infection while ensuring necessary CIED treatment. As reported in previous studies, the timing of reimplantation may affect the risk of device reinfection.^33^ In our study, no significant difference in the rate of recurrent bacteraemia with IE was observed when comparing patients reimplanted within 14 days vs. later. The overall number of events was small in both groups, indicating a generally low risk of reinfection and no evidence of increased risk associated with earlier reimplantation in selected patients. A randomized clinical trial (POET-PM) is currently underway to address this very question (ClinicalTrials.gov identifier: NCT06250985).

### Factors associated with reimplantation

Our findings align with previous studies,^27^ which indicate that atrioventricular block (AVB2/3) and atrial fibrillation as a comorbidity are significant factors associated with CIED reimplantation—likely due to the critical nature of these conditions,^34,35^ especially AVB2/3 as a pacing indication. In contrast, SSS was significantly associated with a lower rate of reimplantation. This trend is consistent with prior research, suggesting that clinicians may be more cautious about reimplantation when pacing needs are less urgent. Overall, the concordance between our results and earlier studies underscores the notion that the severity of the underlying conduction disorder plays a pivotal role in reimplantation decisions. Although the higher prevalence of reimplantation in men compared with women lost statistical significance after adjustment, this finding aligns with previous studies showing that women are generally less likely to receive device implantation overall.^36–39^ The lack of a significant association between ICD and reimplantation is noteworthy, given that ICD recipients often have severe underlying cardiac disease, which would typically suggest a strong need for reimplantation. However, some may transition to palliative care, thereby reducing reimplantations. Although age >80 years was associated with a higher prevalence of reimplantation, the age itself alone does not imply a palliative approach. This association may reflect a selection bias, as only elderly patients with sufficient functional status and life expectancy would have been considered for reimplantation, whereas frailer patients may have transitioned to palliative care. Furthermore, this finding may also be related to differences in device indication, as pacing indications such as second- and third-degree AV block were more common among patients over 80 years. Others may experience improved left ventricular function or decreased arrhythmic risk post-extraction, obviating the need for, or the strength of the indication for a new ICD. In a supplementary analysis restricted to patients with an ICD or CRT-D, secondary prevention ICDs were significantly more frequent in the reimplantation group suggesting that indication type may influence reimplantation decisions in clinical practice. We also observed that patients who did not undergo reimplantation had a higher frailty score, highlighting the complexity of managing comorbid populations where the risks of reimplantation may outweigh its benefits.^30^

Furthermore, our data indicate a low rate of IE readmissions and reinfection in both the reimplantation and non-reimplantation groups, aligning with other studies.^5^ This supports a selective approach to reimplantation without substantially increasing the risk of infection. Nevertheless, we observed higher mortality in the non-reimplantation group, although the difference was not statistically significant. This finding is consistent with previous studies.^27,40^ One study^40^ reported that the majority of deaths were non-cardiac and unrelated to the absence of a CIED, thereby reinforcing the theory that increased mortality in the non-reimplantation group may be driven by higher frailty, greater comorbidity burdens, and a poorer overall prognosis, which may contribute to selection bias.

### Strength and limitations

A major strength of this study is the use of high quality, nationwide registry-based data, which allows for accurate patient identification and outcome assessment across multiple registries. The inclusion of validated diagnostic and procedural codes with high PPVs for both IE diagnoses and CIED extraction codes, as shown in previous Danish validation studies^15,17^ including the NIDUS design study,^41^ supports the accuracy of case ascertainment. This, along with complete nationwide coverage and no loss to follow-up, enhances the reliability and generalizability of our findings.

However, several limitations should be acknowledged. First, the observational study design inherently limits causal inference. Second, despite adjusting for potential confounders, residual confounding may persist due to the absence of detailed clinical data, including the classification of IE (e.g. left-sided vs. CIED infection, and lead vs. pocket infection), precise CIED indication, antibiotic treatment strategies, imaging results, microbiological findings from extracted devices, data on extraction completeness (i.e. complete vs. incomplete extraction), peri- or post-procedural complications, clinical decision-making processes, and patient preferences. Additionally, we did not have access to data on temporary pacemaker use after extraction, including the proportion of patients requiring temporary pacing or the duration of support. Furthermore, data on pacemaker interrogation or telemetry findings before and after extraction were not available, preventing a comprehensive assessment of pacing requirements. Although the risk of misclassification is likely minimal given the high PPVs, it cannot be fully excluded, particularly for differentiating possible from definite CIED-related infections. Lastly, the timing analysis is limited by the small number of patients reimplanted within 14 days and by the clinical decision-making that inherently influences the timing of reimplantation.

## Conclusion

In this nationwide study of patients with IE who underwent CIED extraction without valve surgery, we found that ∼60% of the study population underwent CIED reimplantation within 3 months following extraction, with a median time to reimplantation of 29 days. Second- and third-degree AVB, atrial fibrillation, and age over 80 years were associated with significantly higher reimplantation rates. The cumulative incidences of IE readmissions, recurrent bacteraemia, and all-cause mortality were low with no significant difference by reimplantation status or timing. We also observed a transition from dual- to single-chamber pacemakers following reimplantation. Our findings highlight the complexity of CIED reimplantation decisions, emphasizing the importance of individualized patient evaluation to optimize clinical outcomes. Future research should focus on strategies to balance the risks of early vs. delayed reimplantation and strategies to balance the risk of reimplantation vs. no reimplantation.

## Supplementary Material

oeag008_Supplementary_Data

## Data Availability

The data in this study are provided by Statistics Denmark. Although the data are not publicly available due to Danish data protection regulations, access can be granted to authorized researchers following approval from the relevant authorities.
