# Supplementary material for: Extraction and reimplantation of cardiac implantable electronic devices in patients with non-surgically treated infective endocarditis: a nationwide cohort study
Source: Eur Heart J Open. 2026 Jan 21;6(1):oeag008. doi: 10.1093/ehjopen/oeag008 (PMC12930842; doi:10.1093/ehjopen/oeag008)
Supplement: oeag008_Supplementary_Data [file oeag008_supplementary_data.docx]

**Supplementary data:**

**Figure S1:** Bar chart - Distribution of bacterial groups by reimplantation status.

*
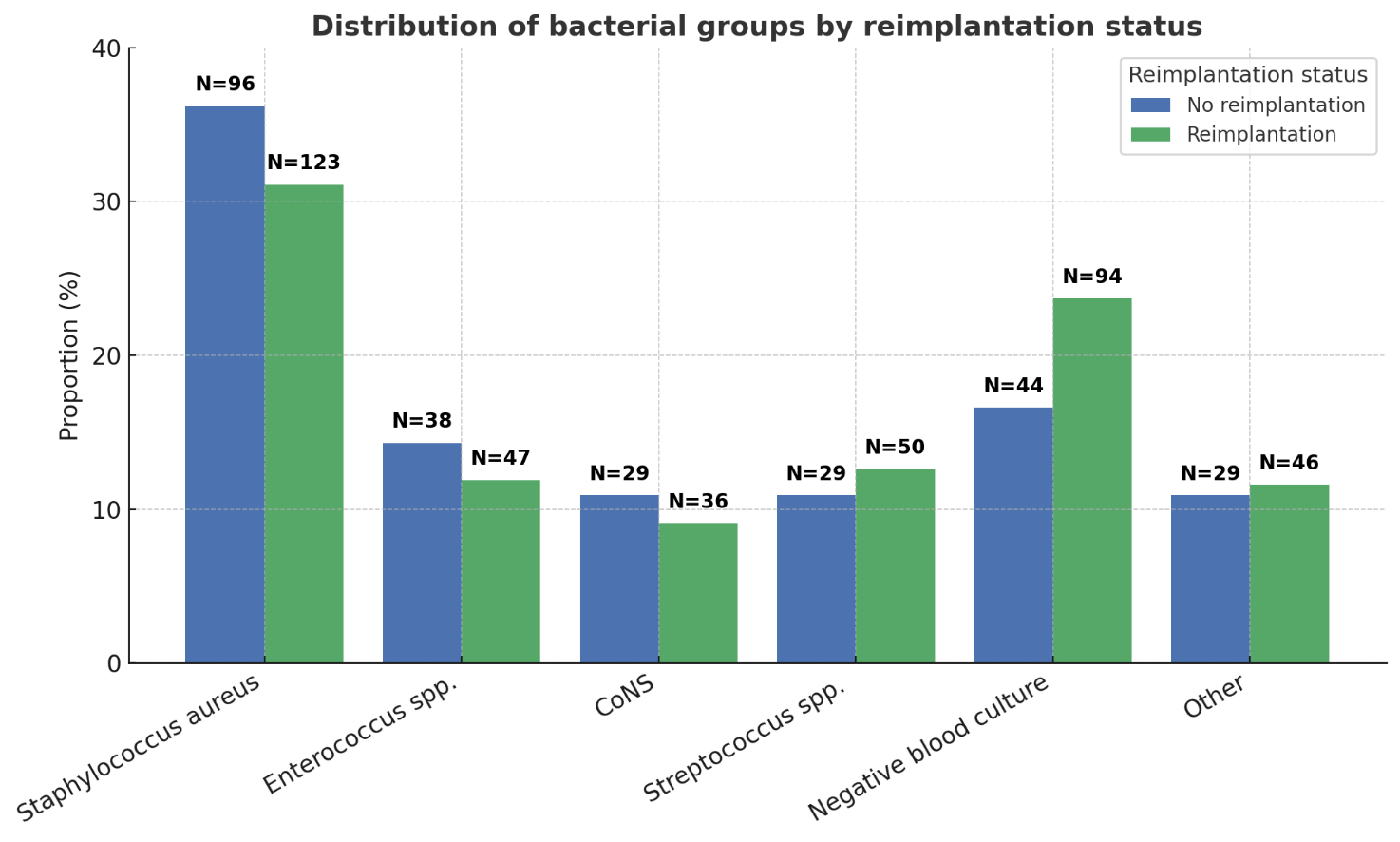
CoNS, coagulase-negative staphylococci.*

**Figure S2:** Cause-specific Cox regression model**;** Factors associated with CIED reimplantation within 90 days

**
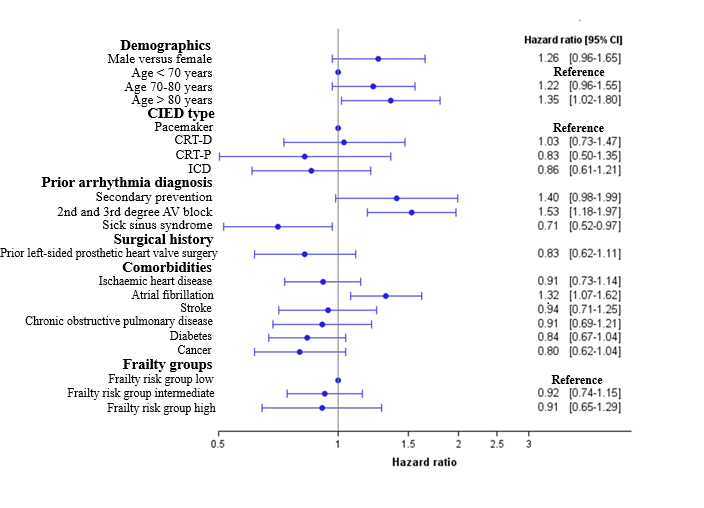
**

******* *Hazard ratios (HR) with 95% confidence intervals (CI) are shown. Error bars represent 95% CI. Estimates are adjusted for the variables shown as well as calendar year and time since most recent CIED implantation.*

**Table S1: NCSP codes for procedures**

| **Procedure** | **NCSP codes** |
| --- | --- |
| **PM** | BFCA0, BFCA1, BFCA2, BFCA01, BFCA02, BFCA03, BFCA07, BFCA20, BFCA25 ¨ |
| **ICD** | BFCB0, BFCB2, BFCB5, BFCB00, BFCB01, BFCB02, BFCB20, BFCB25, BFCB50, BFCB51, BFCB52 |
| **CRT-P** | BFCA04, BFCA05, BFCA06, BFCA21 |
| **CRT-D** | BFCB03, BFCB21, BFCB53 |
| **Heart valve surgery** | KFK, KFM, KFG, KFJ |
| **Left-sided prosthetic heart valve surgery** | KFKD, KFMD |

******* *Hazard ratios (HR) with 95% confidence intervals (CI) are shown. Error bars represent 95% CI. Estimates are adjusted for the variables shown as well as calendar year of IE admission and time since most recent CIED implantation.*

**Table S2: NCSP codes for extraction of cardiac implantable electronic device**

| **CIED extraction:** | **NCSP codes** |
| --- | --- |
| Lead extraction/explantation | BFCA8, BFCA80, BFCA81, BFCA82,  BFCB8, BFCB80, BFCB81, BFCB82 |
| Removal of PM/ICD | BFCA4, BFCB4, KFPH |

*ICD, Implantable cardioverter defibrillator; NCSP, Nordic Medico-Statistical Committee Classification of Surgical procedures; PM, Pacemaker*

**Table S3: Comorbidities, ICD-8 and - 10 codes**

| **Comorbidities** | **ICD-8 codes** | **ICD-10 codes** |
| --- | --- | --- |
| **Ischemic heart disease** | 410-414 | I20-I25 |
| **Heart failure** | 425, 428, 4270, 4271 | I42, I50, I110, I130, I132, J819 |
| **Atrial fibrillation** | 42793, 42794 | I48 |
| **Stroke** | 430-434, 436 | I60-164 |
| **Diabetes** | 250 | E10-E14  or *ATC code*: A10 |
| **COPD** | 490-492 | J42-J44 |
| **Cancer** | 140-209 (if not 173) | C00-C97 (if not C44) |
| **Chronic renal disease** | 403, 404, 581-584, 25002, 40039, 59009, 59320, 75310, 75311, 75319 | E102, E112, E132, E142, I120, N02-N08, N11, N12, N14, N18, N19, N26, N158-N160, N162-N164, N168, M300, M313, M319, M321B, Q612, Q613, Q615, Q619, T858, T859, Z992 |
| **Liver disease** | 070, 155, 571-573 | B15-B19, C22, D684C, K70-K77, I982, Q618A, Z944 |

*COPD, chronic obstructive pulmonary disease;
ICD-8, 8^th^ revision of the International Classification of Diseases; ICD-10, 10^th^ revision of the International Classification of Diseases*

**Table S4. Pharmacotherapy, ATC classification codes**

| **Pharmacotherapy** | **ATC codes** |
| --- | --- |
| **Beta blockers** | C07, C09BX |
| **Calcium channel blockers** | C07F, C08, C09BB, C09DB |
| **Renin angiotensin system inhibitors** | C09 |
| **Thiazide** | C03A, C03EA01, C07B, C07D, C09XA52 |
| **Loop diuretics** | C03C, C03EB01, C03EB02 |
| **Spironolactone** | C03DA01, C03DA02, C03DA03, C03DA04 |
| **Digoxin** | C01AA05 |
| **Cholesterol lowering drugs** | C10 |
| **Antidiabetics** | A10 |
| **Acetylsalicylic acid** | B01AC06 |
| **Anticoagulants** | B01AA, B01AE, B01AF |
| **Hypertension** | Adrenergic alpha-antagonists (C02A, C02B, C02C), vasodilators (C02DB, C02DD, C02DG), loop diuretics, beta blockers, calcium channel blockers, thiazide, spironolactone and renin-angiotensin system inhibitors |
| **Immunosuppressive medication** | L04 |

*ATC, Anatomical Therapeutic Chemical.*

**Table S5: NCSP codes for reimplantation of cardiac implantable electronic devices**

| **CIED reimplantation** | **NCSP codes** |
| --- | --- |
| PM | BFCA1, BFCA10, BFCA11, BFCA12, BFCA0, BFCA01, BFCA02, BFCA03, BFCA07, BFCA08 |
| ICD | BFCB0, BFCB00, BFCB01, BFCB02, BFCB04, BFCB5, BFCB50, BFCB51, BFCB52 |
| CRT-P | BFCA2, BFCA20, BFCA21, BFCA25, BFCA04, BFCA05, BFCA06 |
| CRT-D | BFCA2, BFCA20, BFCA21, BFCA25, BFCB03, BFCB53 |

*ICD, Implantable cardioverter defibrillator; NCSP, Nordic Medico-Statistical Committee Classification of Surgical procedures; PM, Pacemaker*
